# Supplementary material for: Colonisation with extended spectrum beta-lactamase-producing and carbapenem-resistant Enterobacterales in children admitted to a paediatric referral hospital in South Africa
Source: PLoS One. 2020 Nov 6;15(11):e0241776. doi: 10.1371/journal.pone.0241776 (PMC7647087; doi:10.1371/journal.pone.0241776)
Supplement: S1 File — (DOCX) [file pone.0241776.s001.docx]

**S1 File: References for ESBL and Carbapenemase PCR**

| **Primer** | **Sequence 5' - 3'** | **Length** | **Reference** |
| --- | --- | --- | --- |
| **CARBAPENEMASES** | | | |
| GesF | ATGCGCTTCATTCACGC | 17 | [1] |
| GesR | GCTCAGGATGAGTTGTG | 17 | [1] |
| ImpXF2 | ATTGACACTCCATTTAC | 17 | [2] |
| ImpXR2 | AACAACCAGTTTTGC | 15 | [2] |
| NdmF2 | GGTTTGGCGATCTGGTTTTC | 20 | [3] |
| NdmR2 | CGGAATGGCTCATCACGATC | 20 | [3] |
| Oxa48F | CGTGTATTAGCCTTATCG | 18 | [3] |
| Oxa48R | CGCTAACCACTTCTAGG | 17 | [3] |
| VimXF | GTGAGTATCCGACAGTC | 17 | [2] |
| VimXR | GAGCAAGTCTAGACCG | 16 | [2] |
| KpcF | TGTCACTGTATCGCCGTC | 18 | [4] |
| KpcR | CTCAGTGCTCTACAGAAAACC | 21 | [4] |
| **ESBL’s** | | | |
| CTXM1F | CGCTTTGCGATGTGCAG | 17 | [1] |
| CTXM1R | ACCGCGATATCGTTGGT | 17 | [1] |
| SHVC | AGAAGGGTTATTCTTATTTGTCGC | 24 | [5] |
| SHVD | TCTTTCCGATGCCGCCGCCAGTCA | 25 | [5] |
| DEB | ATGAGTAAACTTGGTCTGAC | 20 | [6] |
| 3061TEM | AGGAAGCAAAGCTGAAAGGAATCAAATTTGG | 31 | [1] |

New Delhi Metallo-beta-lactamase (NDM), *Klebsiella pneumoniae* carbapenemase (KPC), Oxacillinase-48 (OXA-48) and variants, Imipenemase (IMP), Verona Integron-Mediated Metallo-beta-lactamase (VIM) and Guiana extended spectrum carbapenemase (GES).

Temoneira (TEM), cefotaxime (CTX), cefotaxime (CTX)-M-type (CTX-M), *s*ulf*h*ydryl *v*ariable (SHV)

**References**

1. Segal H, Elisha BG. Resistance to beta-lactams, and reduced susceptibility to carbapenems, in clinical isolates of *Klebsiella pneumoniae* due to interplay between CTX-M-15 and altered outer membrane permeability. The South African Journal of Epidemiology and Infection. 2006;21(2):41-4.

2. Jacobson RK, Minenza N, Nicol M, Bamford C. VIM-2 metallo-beta-lactamase-producing Pseudomonas aeruginosa causing an outbreak in South Africa. J Antimicrob Chemother. 2012;67(7):1797-8. doi: 10.1093/jac/dks100. PubMed PMID: 22457310.

3. Manesen R, Bamford C, Smith M, Jacobson R, Moodley C, Williams S. Outbreak of carbapenem-resistant *Klebsiella* sp. in an academic hospital, Western Cape, May 2012. NationaI Institute for Communicable Diseases Surveillance Bulletin. 2012;10(4):82-6.

4. Yigit H, Queenan AM, Anderson GJ, Domenech-Sanchez A, Biddle JW, Steward CD, et al. Novel carbapenem-hydrolyzing beta-lactamase, KPC-1, from a carbapenem-resistant strain of Klebsiella pneumoniae. Antimicrob Agents Chemother. 2001;45(4):1151-61. doi: 10.1128/AAC.45.4.1151-1161.2001. PubMed PMID: 11257029; PubMed Central PMCID: PMCPMC90438.

5. Wang H, Kelkar S, Wu W, Chen M, Quinn JP. Clinical Isolates of Enterobacteriaceae Producing Extended-Spectrum β-Lactamases: Prevalence of CTX-M-3 at a Hospital in China. Antimicrobial Agents and Chemotherapy. 2003;47(2):790-3. doi: 10.1128/aac.47.2.790-793.2003.

6. Canica MM, Lu CY, Krishnamoorthy R, Paul GC. Molecular diversity and evolution of blaTEM genes encoding beta-lactamases resistant to clavulanic acid in clinical E. coli. J Mol Evol. 1997;44(1):57-65. PubMed PMID: 9010136.
